# Highly Efficient F, Cu doped TiO_2_ anti-bacterial visible light active photocatalytic coatings to combat hospital-acquired infections

**DOI:** 10.1038/srep24770

**Published:** 2016-04-21

**Authors:** Nigel S. Leyland, Joanna Podporska-Carroll, John Browne, Steven J. Hinder, Brid Quilty, Suresh C. Pillai

**Affiliations:** 1 Kastus Technologies, 5 Fitzwilliam Square East, Dublin 2, Ireland; 2 Centre for Research in Engineering Surface Technology (CREST), DIT FOCAS Institute, Kevin St, Dublin 8, Ireland; 3 The Surface Analysis Laboratory, Faculty of Engineering and Physical Sciences, University of Surrey, Guildford, Surrey, GU2 7XH, United Kingdom; 4 School of Biotechnology, Dublin City University, Dublin 9, Ireland; 5 Nanotechnology Research Group, Department of Environmental Sciences, Institute of Technology Sligo, Ireland; 6 Centre for Precision Engineering, Materials and Manufacturing Research (PEM), Institute of Technology Sligo, Sligo, Ireland

## Abstract

Bacterial infections are a major threat to the health of patients in healthcare facilities including hospitals. One of the major causes of patient morbidity is infection with *Staphylococcus aureus*. One of the the most dominant nosocomial bacteria, Methicillin Resistant *Staphylococcus aureus* (MRSA) have been reported to survive on hospital surfaces (*e.g*. privacy window glasses) for up to 5 months. None of the current anti-bacterial technology is efficient in eliminating *Staphylococcus aureus*. A novel transparent, immobilised and superhydrophilic coating of titanium dioxide, co-doped with fluorine and copper has been prepared on float glass substrates. Antibacterial activity has demonstrated (by using *Staphylococcus aureus*), resulting from a combination of visible light activated (VLA) photocatalysis and copper ion toxicity. Co-doping with copper and fluorine has been shown to improve the performance of the coating, relative to a purely fluorine-doped VLA photocatalyst. Reductions in bacterial population of log_10_ = 4.2 under visible light irradiation and log_10_ = 1.8 in darkness have been achieved, compared with log_10_ = 1.8 under visible light irradiation and no activity, for a purely fluorine-doped titania. Generation of reactive oxygen species from the photocatalytic coatings is the major factor that significantly reduces the bacterial growth on the glass surfaces.

The issue of healthcare acquired infections (HCAI) is of major concern across the globe^1^,^2^,^3^,^4^,^5^. The hospital environment is an important but often neglected factor in hospital cross-infection^6^ Data from the 2014 report of the European Centre for Disease Prevention and control showed resistance to methicilin in *Staphylococcus aureus* samples taken from hospitals at rates greater than 10% in in 19 out of 29 EEA countries. Resistance rates were 17.4% in France, 11.3% in Germany, 11.8% in the UK and as high as 56.0% in Romania^7^. Many studies have shown that these organisms can contaminate surfaces and equipment touched by hospital staff 8,9 including furniture and medical equipment^10^,^11^. In one study 92.9% of writing tools taken from 42 doctors showed bacterial contamination^12^. It is likely that the doctors in question adhered to appropriate hygiene practices but that fomites (inanimate objects that can harbour and serve in transmitting bacteria), such as door handles, table tops and any other contactable surfaces held bacterial reservoirs for transfer. Two of the most prevalent nosocomial bacteria, Methicillin Resistant *Staphylococcus aureus* (MRSA) and *Clostridium difficile* (*C. diff* ), have been shown to survive on such surfaces for up to 5 and 7 months respectively^7^. *C. diff* spores in particular are highly resistant to alcohol based cleaning and disinfection solutions^13^. As surface contamination can contribute to transmission of bacteria^14^, the development of surface enhanced coatings that causes sanitisation of the surface is an important development in reducing hospital acquired infections.

The use of photocatalysts for hygiene applications has been the subject of recent research, but is not a common practice as normally a high level of UV exposure is required to activate the catalysts^15^,^16^,^17^,^18^.

Existing hygiene coatings based on photocatalysts suffer from two principal drawbacks. Firstly, most rely on ultraviolet light to generate free electrons and subsequent reactive species. This limits their use to situations with a significant UV flux, typically relying on sunlight or else requiring deliberate exposure to artificial UV light sources, the latter having the obvious disadvantage of compromising human wellbeing. Secondly, a purely photocatalytic hygiene coating is inactive in darkness, apart from the very short time after exposure, during which the population of reactive species rapidly declines.

Previous studies have led to the development of VLA titania photocatalysts that form transparent conformal films, by the deposition of a precursor sol and subsequent heat treatment to crystallise the titania and fuse it to the substrate surface^19^,^20^.

It has been known for some time that several transition metals are toxic to a range of pathogenic microbial species and this has informed both widespread research on the use of such materials as practical antimicrobial agents and considerable commercial activity in this area. Work has been focussed principally on copper and silver as the active agents. While silver has been shown to be toxic to a wide range of pathogens^21^,^22^, with limited impact on human health, financial concerns have restricted its use to a minority constituent at low concentrations, principally as nanoparticle dispersions or a substituent for ion release from an inert and less costly matrix. Silver-substituted zeolites offer an attractive delivery vehicle, having a large surface area for controlled ion release, while requiring a low silver content and being easily dispersible^23^. Copper, its compounds and alloys are also the subject of considerable attention, both as dispersions and in the case of elemental copper and its alloys, for bulk fabrication and cladding^24^,^25^,^26^. In all form factors, copper and copper alloys have a considerable cost advantage over silver, particularly for fabrication, where the cost of silver is prohibitive^27^,^28^.

This paper reports the successful application of a visible light activated (VLA) anatase titanium dioxide photocatalyst to control and reduce populations of pathogenic bacteria.

This coating incorporates both a VLA photocatalyst and a metal ion release mechanism to ensure that it remains bactericidal in both light and darkness and is formed in a single deposition and heat treatment process. The copper ion release does not adversely affect the photocatalytic efficiency of the titanium dioxide and has only a limited impact on the transparency of the coating.

## Materials and Methods

### Materials and reagents

Titanium isopropoxide (purity > 97%), glacial acetic acid (>99.7%), trifluoroacetic acid (99%),) and. Copper (II) nitrate pentahemihydrate (98%) were obtained from Sigma Aldrich. All of the materials were used without any further purification.

### Sol Synthesis

An aqueous sol of titanyl acetate and titanyl trifluoroacetate, optionally doped with copper nitrate was prepared according to the method of US Patent 9,210,934^29^ and UK Patent GB2521405^30^.

In order to prepare the formulation, glacial acetic acid (24 mL) was added to a glass beaker while continuously stirring at room temperature. Next, titanium isopropoxide (12.5 mL) was added slowly in a drop-wise manner, and the mixture allowed to continue stirring for a period of 30 minutes. Then trifluoroacetic acid (4 mL) was added drop-wise and the solution was left to stir for 10 minutes. To prepare a VLA titanium dioxide precursor sol, 150 ml of water was added slowly to the previously prepared solution. At this stage a transparent, colourless solution was obtained which was left to mix for another 30 minutes. In order to remove any remaining agglomerates, the obtained formulation was filtered using 0.22 μm syringe filter and stored in the fridge prior to coating on a substrate.

To prepare a copper-doped VLA titanium dioxide precursor sol, glacial acetic acid (24 mL), titanium isopropoxide (12.5 mL) trifluoroacetic acid (4 mL) were mixed according to the protocol above. Meanwhile, the copper precursor, copper (II) nitrate pentahemihydrate (1.393 g) was added to water (150 ml), completely dissolved and then added slowly to the previously prepared solution. At this stage a transparent, blue coloured solution was obtained which was left to mix for another 30 minutes. This formulation was also filtered using 0.22 μm syringe filter and stored in the fridge prior to coating on a substrate.

### Film Deposition and Crystallisation

The sol was deposited on a cleaned glass Plate, 50 mm × 55 mm, by means of dip coating. Substrates were immersed in a vessel of the sol at room temperature, then withdrawn vertically at a rate of 1.45 mms^−1.^

The coated glass was allowed to dry at room temperature for 30 minutes at room temperature, before being transferred to the furnace, and then heated for 90 minutes at 550 °C, to complete the transformation of the mixed titanyl acetate- titanyl trifluoroacetate precursor gel to titanium dioxide, its crystallisation to the anatase structure and the simultaneous partial substitution of fluorine for oxygen and in the copper-doped sols, copper for titanium, to achieve the desired doping.

After heat treatment, a 5 mm wide strip was cut from the edge that had been gripped by the sample holder during dip coating, leaving a fully coated 50 mm × 50 mm coupon for microbiological testing.

### Microbiological Testing

Gram-positive *Staphylococcus aureus* (ATCC 6538) was chosen to conduct the pure culture studies. In order to prepare cultures for testing, 10 ml of sterile nutrient broth were inoculated with colonies of *S.aureus* and incubated overnight at 37 °C without shaking. Obtained stock cultures were centrifuged and washed with phosphate buffer saline (PBS) twice (15 min at 4,000 rpm). The concentration was adjusted accordingly to approx. 10^6^ cfu/sample based on the calibration curve prepared for the organisms.

**Figure 1:**
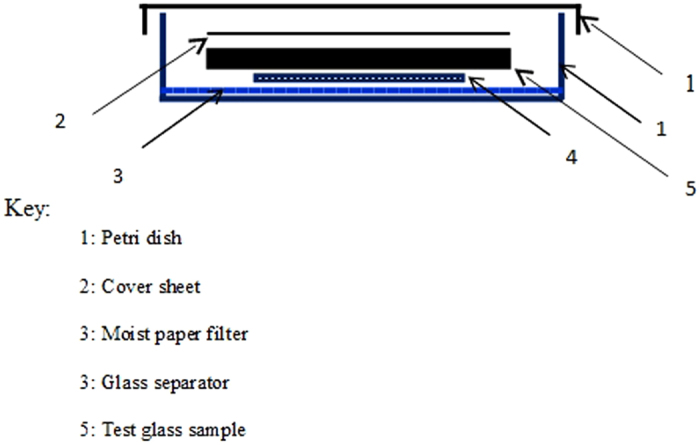
Experimental setup – The “wet chamber”according to ISO27447. (**1**) Glass Petri dish, (**2**) Cover sheet, (**3**) Moist paper filter, (**4**) Glass separator, (**5**) Test glass sample.

For the testing, each glass sample was aseptically placed in a sterile Petri dish containing moist filter paper. This was prepared by adding a sterilised moisture control paper filter in the bottom of a sterilised Petri dish. An adequate amount of sterile water was added to the paper filter (approximately 3–4 ml water). A glass plate was inserted in order to avoid contact between the test piece and the filter paper (Fig. 1). Next, 75 μl quantities of the adjusted bacterial suspensions (approx. 1 × 10^5^ CFU/sample) were aseptically dispensed onto the centre of each sample and covered with transparent polymer film. The prepared samples were divided into two groups (test samples and controls). One of each was immediately placed in a cardboard box with cover (dark) and another set were exposed to T5 lighting (1,000 lux) for 24 hours at room temperature. For each set, the test was performed in triplicate.

Following 24 hour of incubation, all samples were processed to analyse for any viable bacteria remaining on the surface, post-exposure. Film was removed from sample surface first and aseptically transferred to a sterile stomacher bag containing 10 ml of sterile PBS. This was rubbed in order to remove remaining organisms from the surface. The test glass sample was then placed in the same stomacher bag and the same procedure was repeated. Serial dilutions were performed on the extraction solution accordingly. 1 ml aliquots of each dilution as well as the neat extraction solution were aseptically dispensed into sterile Petri dishes in duplicate. Approximately 15 ml of molten plate count agar was poured into each plate swirled and allowed to solidify. All plates were incubated aerobically at 37 °C for 24 hours after which a colony count was performed.

### X-Ray Diffraction

Samples of the sol were dried at 120 °C, and then calcined in air at temperatures between 300 °C and 600 °C. X-Ray diffractometry was performed on the calcined powders, using a Siemens D500 diffractometer employing Cu-Kα radiation.

### Optical Transmission

The optical transmission of heat treated films on glass was measured, using a Perkin Elmer Lambda 900 UV/Visible/NIR spectrometer over a wavelength range of 600 nm to 200 nm.

Optical absorbance measurements were performed on mixtures of doped titania powders, formed by calcination at 330 °C and 550 °C at a concentration 0.5% in potassium bromide, compressed into transparent discs, using a Shimadzu 1600 UV/Visible/NIR spectrometer over a wavelength range of 600 nm to 200 nm. From these spectra, the band gap potential was calculated from the intercept tangents to the spectrum in the visible range and the transitional range from visible light transparency to ultraviolet opacity.

### X-Ray Spectroscopy

XPS analyses were performed with a Thermo VG Scientific ESCALAB Mk II spectrometer and Alpha 110 analyser, using Al Kα radiation (hν = 1486.6 eV) at 300 W. All spectra were corrected for charging by reference to the C 1 s peak at 285 eV.

### Surface Wetting and Contact Angle Measurement

The hydrophobic or hydrophilic nature of the surfaces of coated and uncoated substrates was characterised by means of a liquid contact angle measurement. Measurements were performed using a First Ten Angstroms Model 200 system and the wetting liquid was glycerol. Three measurements were performed on each surface and a mean calculated. The contact angles of the lower surface of the substrate that had been in contact with the tin bath during manufacturing and of the upper surface that had been exposed to air was measured separately.

## Results and Discussion

Suitable choices of dopant elements and their concentration has enabled coatings to be developed in which the anatase to rutile transformation can be suppressed at temperatures of up to 900 °C^31^,^32^, allowing the application of VLA photocatalytic coatings to a wide range of substrates during existing manufacturing processes. Fluorine is an attractive dopant, since it has been found both to confer visible light activity and to stabilise anatase at elevated temperatures, thus fulfilling both roles with a single modification to the synthesis^20^,^31^. Addition of F is effective in titania doping as it can minimize the recombination of the electron hole pair due to charge compensation between F^-^ and Ti^4+ ^33. In addition F-doping is reported to induce the creation of Ti^3+^ ions and thereby oxygen vacancies^33^.

### X-Ray Diffraction

**Figure 2:**
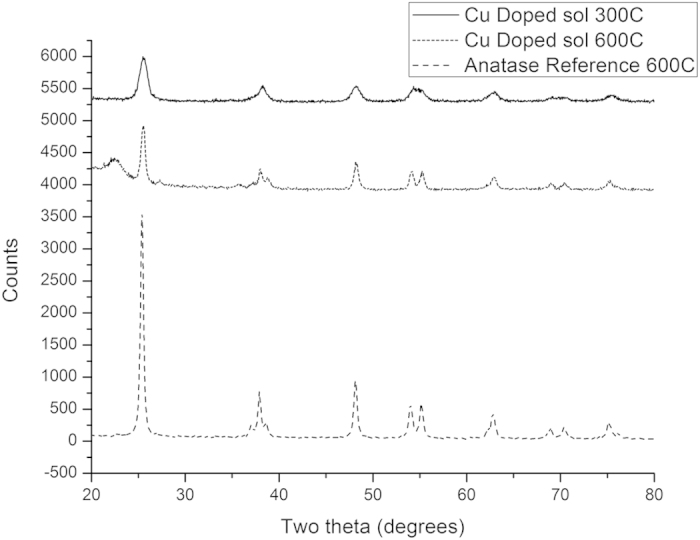
XRD Spectra of Anatase Reference Sample and Copper Doped Titania Calcined at 300 °C and 600 °C.

X-Ray diffraction spectra obtained from powders calcined at 300 °C and 600 °C are shown in Fig. 2, together with the spectrum of an anatase titania reference sample. As can be seen in Fig. 2, calcination at both temperatures results in the formation of a largely crystalline product, with a diffraction pattern characteristic of anatase. The peaks are relatively broad and less well differentiated than those in the reference spectrum, particularly at 300 °C, which we attribute to a combination of incomplete crystallisation at lower temperatures and peak spreading caused by the nanoparticulate nature of the material. Inhibition of titania condensation and crystallisation in fluorine-rich doped systems has already been noted by other workers^31^.

There are no additional peaks that could be attributed to copper oxides and from this we conclude that much of the copper is dissolved in the titania matrix, with any copper oxide being present at concentrations below the detection level of the technique.

### Optical Transmission

**Figure 3:**
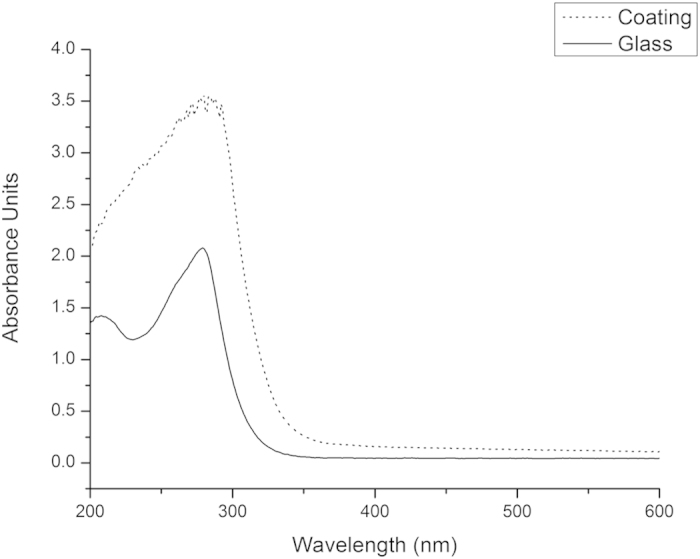
Optical Absorbance Spectra of Uncoated and Coated Glass.

The optical absorbance spectra of the glass substrate and glass coated with a two layer copper doped coating are shown in Fig. 3, below.

It can be seen that while the coating reduces the overall transparency of the glass, the absorbance spectrum in the visible range is still essentially flat, so the coated substrates do not have a colour cast. Absorption in the coated materials increases above that of the substrate at wavelengths below approximately 380 nm and rapidly increases at wavelengths below 300 nm, consistent with absorption of blue and particularly of ultraviolet light by the titanium dioxide.

### Surface Wetting and Contact Angle Measurements

The results of the contact angle measurements are shown in Table 1, below.

Relatively little difference in contact angle was seen between the two sides of the uncoated glass, though the surface that had been in contact with the tin bath had a marginally higher contact angle (56.46°) than the upper surface that had not been exposed to the tin (52.34°).

The application of the titanium dioxide coating significantly reduced the contact angle to a mean value of 8.51° (superhydrophilic nature).

**Figure 4:**
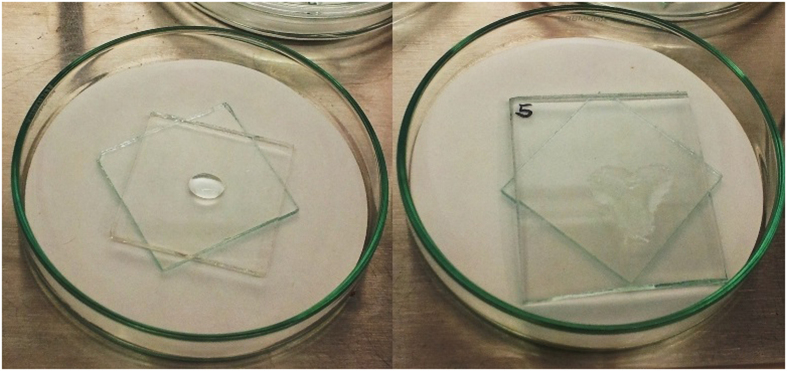
Behaviour of Water Droplet on Coated and Uncoated Glass, Illustrating Superhydrophilic Nature of the Doped Titanium Dioxide Coating. Left side – control, right side – coated glass.

The reduction in contact angle on the application of the titanium dioxide coating is illustrated by the change in macroscopic wetting behaviour of water droplets applied by pipette as sen in Fig. 4. The droplet placed on the uncoated glass displays modest spreading over the surface, while that on the titanium dioxide layer spreads extensively. This is consistent with results found in the literature and is an important factor in the antimicrobial activity of the coating^16^. Photocatalytic degradation by titanium dioxide proceeds principally through the formation of oxidative intermediates, produced by holes in the titania valence band oxidising surface water. Thus, it is advantageous for the surface to be strongly hydrophilic, maximising the opportunity for these reactive peroxide and oxygen radicals to form^16^. The substantial reduction in contact angle also illustrates the importance of obtaining the most uniform coating possible from the first stages of deposition. Since the sol is aqueous and the titania coating is more hydrophilic than the glass substrate, subsequent layers will tend to wet out and adhere preferentially in areas where a partial coating is already present. It is therefore important to ensure that the substrate is uniformly clean and free of contaminants that could increase the water contact angle. Dip coating has been used to prepare the materials in this study, since it ensures complete coverage in a single step.

### Microbiological Testing

The results of microbiological testing of films with *S. aureus* are shown in Table 2, below.

The microbiological testing results show the efficacy of the copper-doped coating both in light and darkness, with at least a substantial reduction in the bacterial population and at best, reducing the bacterial load below detectable levels.

The distinction between the coatings prepared with and without copper doping illustrates the dramatic improvement in performance resulting from the addition of copper to the formulation. The precise degree to which this improved performance is the result of the toxicity of copper to bacteria and how much is potentially caused by increased photocatalytic activity above the solely fluorine-doped material is the subject of ongoing study, but the persistent effectiveness of the copper-doped coatings in darkness implies that the former mechanism is dominant.

In the present study, the negligible photocatalytic activity of undoped titanium dioxide in the absence of ultraviolet radiation is taken as established. This was previously reported in the literature^19^,^33^.

The photocatalytic activity of undoped stoichiometric anatase titania depends on the presence of a significant ultraviolet flux. Previous work by Fisher *et al*.^19^ on photocatlytic activity of titania for water decontamination, in an undoped state and with dopants including copper and nitrogen has demonstrated that the undoped material brings no significant reduction in populations of *E. coli* or *Enterococcus faecalis* in the absence of ultraviolet radiation^19^. In that work, it was found that doping with copper increased the activity slightly, but it was acknowledged that this improved activity could have been caused by a combination of VLA photocatalysis and copper ion toxicity.

### X-Ray Photoelectron Spectroscopy

**Figure 5:**
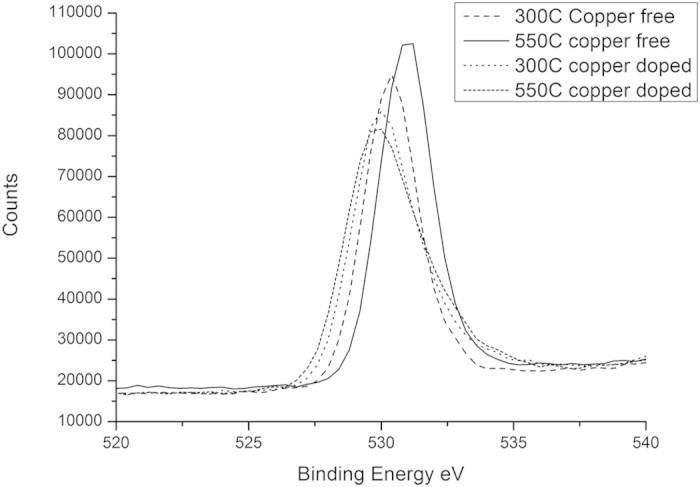
High Resolution Scan of O1s Peak in XPS Spectra.

**Figure 6:**
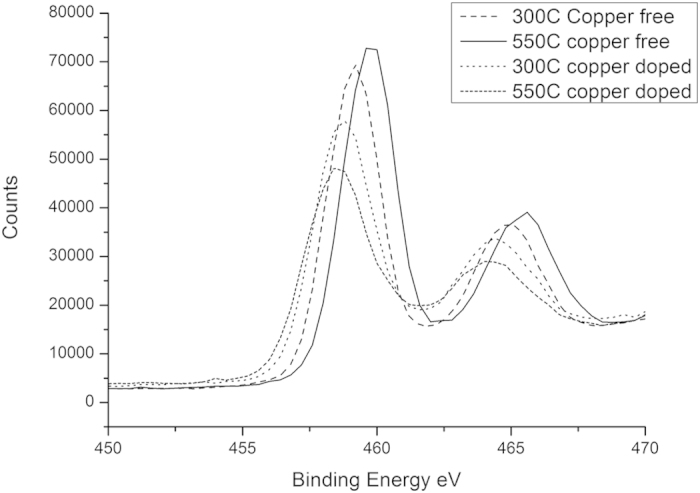
High Resolution Scan of Ti2p Peak in XPS Spectra.

**Figure 7:**
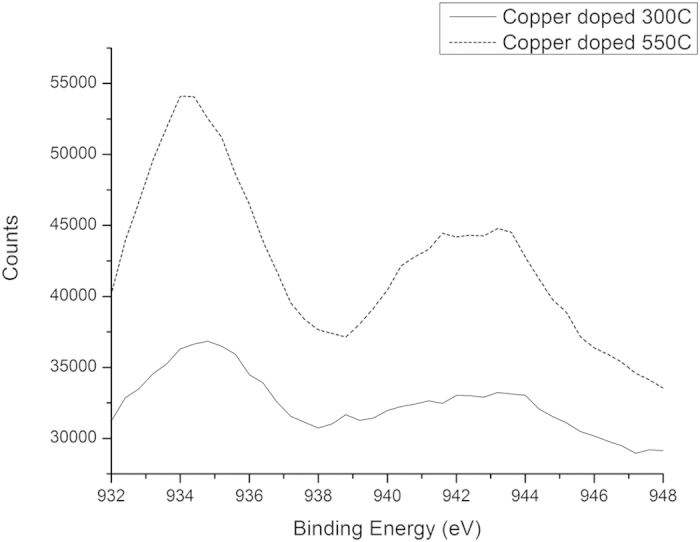
High Resolution Scan of Cu2p Peak in XPS Spectra.

**Figure 8:**
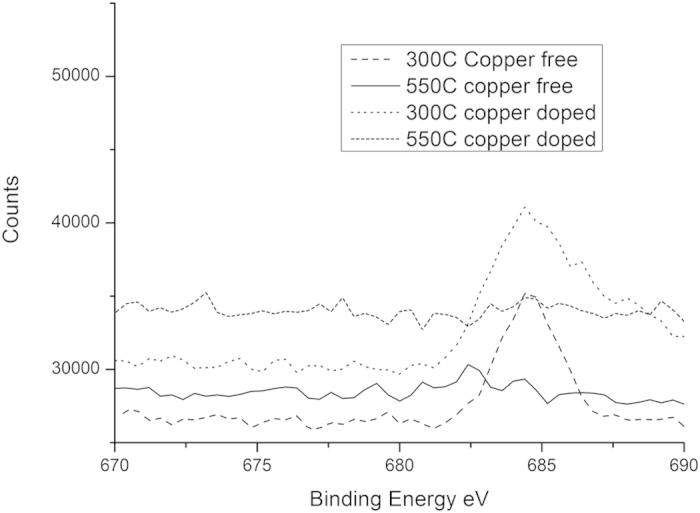
High Resolution Scan of F1s Peak in XPS Spectra.

Selected energy range scans covering the O*1s*, Ti*2p*, Cu*2p* and F*1s* regions of the XPS spectra are shown in figures 5, 6, 7 and 8, respectively.

Addition of copper to the sol substantially modifies the binding energies of both the Ti2*p* and O1*s* electrons in the materials after heat treatment.

As seen in Fig. 5, a consistent downward shift of 0.5 to 0.7 eV in the O*1s* binding energy occurs on the addition of copper to the titanium dioxide matrix. This is consistent the oxygen depletion of the titanium dioxide measured in the composition quantification, with a reduction in oxygen content of 1.5 to 1.8 atomic percent in the co-doped material, relative to that doped with fluorine only. Similar shifts in binding energy were reported by Nolan *et al*.^34^, on the addition of silver to a titanium dioxide matrix. This is consistent with the binding energy of the Cu2p_3/2_ peak in the sample calcined at 300 °C, at 933.74 eV closely matcheing that of CuO, as seen in Fig. 7 35. The absence of any CuO phase detected by XRD suggests incorporation of the copper in the +2 oxidation state into the titanium dioxide matrix, with consequent oxygen depletion of the matrix, relative to stoichiometric titanium dioxide.

The Cu2p_2/3_ binding energy in the copper doped sample heat treated at 550 °C displays an upward peak shift of 0.8 eV to 934.54 eV, greater than the expected deviation in measured values in CuO. This is accompanied by an increase in the oxygen content from 58.54% to 64.69% and is consistent with a super-saturation of oxygen relative to that found in CuO, with a consequent increase in the binding energy caused by the more electronegative environment than would be found in CuO.

The Ti2p binding energy displays downward shifts of 0.6 eV and 0.74 eV in the copper-doped material after treatment at 300 °C and 550 °C respectively, accompanied by a peak broadening of 0.5 eV (Fig. 6), Again, this is consistent with oxygen depletion in the titania and the formation of a minor Ti^2+^ component. Lower Ti2p_3/2_ and O s binding energies for copper doped titania are direct measures of the formation of Ti ^3+^ due to the generation of oxygen^18^. Such a downward shift indicates the existence of oxygen vacancies and Ti ^3+^ in the Cu doped samples. The overall composition is thus oxygen depleted with respect to stoichiometric TiO_2_ and oxygen super-saturated with respect to stoichiometric CuO.

It is also notable that the fluorine content decreases rapidly with heat treatment temperature, in both compositions, falling by factors of 4.51 and 17.87 in the fluorine doped and copper-fluorine co-doped materials respectively. This is illustrated clearly by the near disappearance of the F1s peak in Fig. 8, following heat treatment at 550 °C. After treatment at 550 °C, it can be expected that the properties of the copper-fluorine co-doped material to be dominated by the copper, rather than fluorine doping. This behaviour matches that observed by Padmanabhan *et al*.^31^, in which the fluorine content of titania prepared by the hydrolysis and calcination of a pure titanyl trifluoroacetate was reduced from 0.5 atomic percent after calcination at 550 °C to 0.3 atomic percent after calcination at 700 °C.

**Figure 9:**
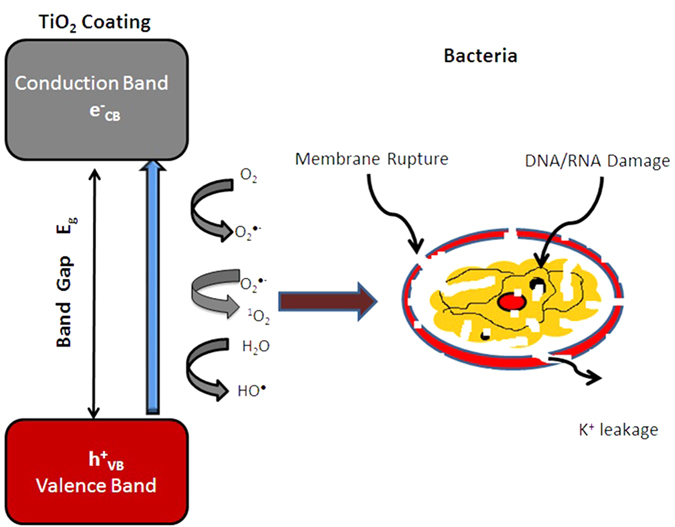
Schematic representation of the photocatalytic anti-bacterial action.

The mechanism for the antimicrobial activity of oxide semiconductor catalysts has been established as cell membrane rupture, caused by reactive oxidising species including hydroxyl radicals and peroxides generated by the oxidation of water and oxygen by holes in the valence band that are created during the photonic excitation of electrons to the conduction band (Fig. 9)^17^.

The superior activity of the copper-doped materials and their continued activity in darkness is the result of a mechanism of a bactericidal action that is independent of that of the photocatalyst, but which has the potential to reinforce it under conditions of illumination.

Copper and its compounds achieve their antimicrobial activity by degrading cell membranes^36^. Previous studies have shown that rapid accumulation of copper ions in *Staphylococcus haemolyticus* was observed on both dry and moist metallic copper surfaces, while no significant mutagenic effects were observed, indicating that absorption of copper does not cause lethal DNA damage, but instead kills by fatally compromising the cell membrane, evidence for which is found from the rapid accumulation of copper ions within the cell, on a scale of minutes. This mechanism is further expanded by Lemire *et al*.^37^ and Macomber *et al*.^38^ who demonstrated that the formation of Cu^2+^ ions is associated with an accelerated formation of reactive oxygen species and this accelerates the consumption of antioxidants, reducing the capacity of the cell wall to absorb damage from these species and to self-repair.

These reactive species are generated by a Fenton reaction as described in Equation 1, thus^39^.


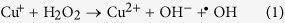


Hydrogen peroxide is a transient metabolic by-product of cellular respiration^37^,^39^, so this mechanism is active whenever cellular respiration is taking place, but it is noteworthy that in the present study, the dissolved copper in the doped titanium dioxide is in a particularly strongly oxidising environment, as indicated by the XPS data, and can thus accelerate the cell wall damage intrinsic to the photocatalytic action.

Similar increases in the bactericidal efficacy of titanium dioxide occurring on the addition of copper have been observed in TiO_2_: Cu films co-sputtered on polyester fabric^40^,^41^. In these experiments, solar spectrum and actinic lights with low UV flux were used. High levels of self-cleaning activity were observed, attributed to the increased generation of reactive oxygen species from a combination of hydroxyl radicals generated by Fenton chemistry^40^ and visible light activated photocatalysis resulting from the presence of intra-band gap energy levels created by the copper doping^41^. Evidence for the effectiveness of copper in inducing VLA photocatalytic activity comes from the substantial increase in decomposition rate of methylene blue in copper doped films, relative to sputtered undoped titanium dioxide under solar spectrum irradiation, where the UV component is relatively minor, which correlated well with a reduction in the estimated band gap^41^.

Closely related systems, also comprising co-sputtered TiO_2_: Cu films have demonstrated antibacterial activity on PET fabric^42^. Evidence for copper-moderated O_2_ reduction as a mechanism for bactericidal activity was provided by the increased activity in aerobic, relative to anaerobic conditions.

Taken together, these results support the argument that copper doping of titanium dioxide increases its photocatalytic and antimicrobial activity by a combination of band-gap modification and hydrogen peroxide splitting through Fenton chemistry.

## Conclusions

A purely fluorine-doped titania displays bactericidal activity under conditions of visible light irradiation, but this is inferior to the activity of a fluorine-copper co-doped material, illustrating the synergistic effect of the combined doping. Co-doping of titanium dioxide with fluorine and copper provides significantly improved antimicrobial activity relative to doping with fluorine alone, both under illumination at visible wavelengths and in darkness. This is attributed to the known toxicity of copper to many species of pathogenic bacteria. The performance of the copper-fluorine co-doped material remains greater under visible light irradiation than in darkness, indicating that the copper does not critically degrade the photocatalytic activity of the titania and we propose that the copper has the potential to increase the yield of highly reactive hydroxyl radicals from photocatalytic oxidation of water. Furthermore, the purely fluorine-doped titania is ineffective in darkness, indicating that its activity is solely photocatalytic.

## Additional Information

**How to cite this article**: Leyland, N. S. *et al*. Highly Efficient F, Cu doped TiO_2_ anti-bacterial visible light active photocatalytic coatings to combat hospital-acquired infections. *Sci. Rep*. **6**, 24770; doi: 10.1038/srep24770 (2016).

## Figures and Tables

**Table 1:** Contact Angles of Glass Substrates and Titanium Dioxide Coatings.

Sample	Glass, tin bath side	Glass, air side	Titania coated glass
Contact angle	56.46°	52.34°	8.51°

**Table 2:** Performance of Fluorine Doped and Copper-Fluorine Doped VLA Titanium Dioxide Coating Against S. aureus Culture.

Sample	% Reduction (light)	% Reduction (dark)	Log Reduction (light)	Log Reduction (dark)
Fluorine doped coating	98.40%	0	1.8	0
Copper – Fluorine doped coating	100.00%	98.30%	4.2	1.8
